# Local control of non-local information flow in oscillatory neuronal networks

**DOI:** 10.1186/1471-2202-12-S1-O15

**Published:** 2011-07-18

**Authors:** Christoph Kirst, Marc Timme, Demian Battaglia

**Affiliations:** 1 Max Planck Institute for Dynamics and Self-Organization, Göttingen, 37073, Germany; 2 Bernstein Center for Computational Neuroscience, Göttingen, 37073, Germany

## 

Control of information flow between neurons or groups of neurons is essential in a functional brain, e.g. for context and brain state dependent processing. In line with recent experimental and theoretical studies [1-5] we show that phase relations between synchronized oscillatory local circuits or brain areas may dynamically create information channels and induce changes in the effective connectivity.

Reducing neuronal oscillatory dynamics to a phase - amplitude description [6,7], we show how alternative phase shifts between different neurons or groups of neurons result in different effective connectivities. In particular, to quantify the information flow, we analytically calculate the time delayed mutual information and transfer entropy between oscillators in a phase locked state. We further present a theoretical framework to predict phase lag patterns within and between groups of oscillators in hierarchical networks. Combining both results we derive the information flow between the oscillators as a function of structural and dynamical network parameter.

We use our results to reveal how effective connectivity is controlled by the underlying physical connectivity and the intrinsic single oscillation frequencies. Interestingly, we find that local changes in the strength of a single link can remotely control the effective connectivity between two different physically unchanged oscillators. Similarly, local inputs modulating the intrinsic frequencies can dynamically and remotely change the information flow between distal nodes.

We link our results to biophysically more realistic networks of spiking neurons. In a clustered network of groups of type I neurons exhibiting gamma oscillations emanating from a PING mechanism [8], we numerically show that local changes of the connectivity or the inputs strengths within a cluster can non-locally control the phase relations and the information flow between distant clusters.

## Conclusion

Our findings reveal that local changes, e.g. in the physical strength of a *local link* or in the *local frequency* due to variation in the *local inputs*, can *remotely and dynamically control* the direction of *non-local global information flow* between distal nodes/clusters in a network. This might provide an efficient local mechanism to control global information processing in neuronal systems and to account for contextual and attentional modulation.
